# Editorial Department

**Published:** 1855-11

**Authors:** 


It is with a feeling of melancholy pride that we give place to the following gazette of the deaths of physicians, in Norfolk and Portsmouth, with the accompanying tribute to their memory from the Virginia Medical and Surgical Journal.

All honor to names like these! Year by year it has been our lot to record the death of faithful soldiers at their posts of danger, battling with the pestilence that walketh at noonday. Ours is a profession of heroes! That cowardice which would shrink from the path of professional duty is unknown among us! We have no renegades! But here at Norfolk, is something nobler even than that sacred impulse which prompts the physician to stay by his home and friends in the hour of danger. Among these forty gallant names are those of many from abroad, from the North, from the far Southwest, and from the inland cities, who have gone down to the fever-smitten coast with their lives in their hands, to offer them up upon the altar of our common humanity. The great world, the noisy crowd which never fails to honor the memory of him who dies upon the battlefield, will pass these names in silence. No funeral pomp, no wailing requiem, no turbid eulogy is theirs, but in the light of that coming morn when the motives of all hearts shall be known, these soldiers of humanity shall outrank and outshine all the gilded heroes of bloody wars!

THE MEMORY OF

| | | | |
|---|---|---|---|
| Sylvester, | Trugien, | Gooch, | Craycroft, |
| Constable, | Parker, | Howle, | Mierson, |
| Halson, | Lovett, | Gelbardt, | Handy, |
| Sylvester, Jr., | Walters, | Blow, | Cole, |
| Higgins, | Thompson, | Jackson, | Morse, |
| Briggs, | Fliess, | De Berane, | Rizer, |
| Upshur, | Booth, | Obermuller, | Smith, |
| Tunstall, | Howe, | De Capry, | Marshall, |
| Selden, | Bache, | Hunter, | Craven, |
| Burns, | Dillard, | Schell, | Berry. |

"At the close of a long and bloody battle, it is the custom to present a list of the killed and wounded; that sad record of the lamented dead, who have gone down to the grave midst the smoke of the conflict; that glorious record of the heroic dead, whose gallant deeds are painted on the pages of history, whose names are cherished in all hearts.

"We, too, have now to tell of like men with these; of some who have fallen at the post of duty; of others who have died whilst serving as volunteers in a deadly campaign. With no hope of victory, with no pomp and circumstance of war to animate the heart, our brethren in Norfolk and Portsmouth have calmly, firmly discharged their duty, and have met their fate. The slaughter is now over, and we record a mortality unprecedented in history.

"Forty physicians have fallen in the hopeless contest. Exhausted with fatigue and watchings; dispirited by their want of success; pressed down with the weight of responsibility resting on them, they have sunk, easy victims to an enemy whose ravages they faithfully labored to resist. Many of these men were residents of the infected cities, and though all was consternation around them, they flinched not at that trying hour, whilst others from all parts of our country, ardently rushed to the scene of danger, and sacrificed their lives in the vain attempt to check the fearful pestilence.

"No pompous funeral accompanied our brethren to their silent grave. No music, sad and mournful, rings upon the ear. They lie quietly now, but they have not died in vain. Faithfully have they fulfilled the sacred duties of their calling, and their memories remain an imperishable legacy to the profession they have ennobled and adorned."

